# Multi-functional voltage and current based enhancement of power quality in grid-connected microgrids considering harmonics

**DOI:** 10.1016/j.heliyon.2024.e26008

**Published:** 2024-02-16

**Authors:** Ehsan Akbari, Abbas Zare Ghaleh Seyyedi

**Affiliations:** a Department of Electrical Engineering, Mazandaran University of Science and Technology, Babol, Iran; b Department of Electrical and Electronics Engineering, Shiraz University of Technology, Shiraz, Iran

**Keywords:** MFGTI, RES, Voltage sag/swell, Harmonic

## Abstract

The introduction of a renewable energy source (RES) based multi-functional grid-tied inverter (MFGTI) stands as a favorable remedy for addressing power quality concerns within distributed generation (DG) systems and microgrids. Nonetheless, the effectiveness of a traditional MFGTI will be restricted in addressing power quality issues based on voltage. The presented research proposes a novel structure for MFGTI to enhance power quality concerns associated with voltage, current, and harmonic distortions resulting from both grid and loads. Based on this strategy, the introduced MFGTI can be linked with the grid by bidirectional switches either in a parallel or series. This feature provides various operational conditions in response to diverse disruptions in the grid. To effectively adjust the voltage, current and voltage reduction are determined through mathematical analysis, considering both the grid conditions and the load requirements. Furthermore, this strategy offers different compensation strategies, control schemes, and transition modes in the MFGTI. The major disturbances such as unbalanced and balanced voltage swell/sag, harmonics, and interruption are compensated. The shunt compensation controller is based on a second order sequence filter (SOSF) to provide the load current active component. A damping PI regulator based series compensation controller is presented for the voltage swell/sag reduction. Moreover, a new three level hierarchical control is proposed in which a droop control for compensating the interruption and a decouple dual synchronous reference frame (DDSRF) for compensating the unbalanced voltage sag/swell are utilized. The simulations in the MATLAB/SIMULINK show that using the proposed compensation strategies, the proposed MFGTI can compensate effectively the different disturbances through changing the transition states by the proposed algorithm based bidirectional switches.

## Highlights

• Shunt connection mode to improve current-based power quality and shallow voltage sag.
• Series connection mode to compensate power quality based on deep voltage sag.
• Compensation of interruption in the loadaccording to the amount of energy stored in MFGTI.
• Harmonic compensation of grid current when the load is nonlinear.
• Providing an algorithm for smooth transition from one mode to another one

## 1 Introduction

Renewable energy sources (RES) account for a key issue in our modern society, however, their integration into the utility grid brings significant technical challenges; the control of RES is among them. Since the versatility of the power grid with the RES could be complex in terms of effectiveness, increasing the reliability, flexibility, and energy efficiency as well as the smart grids’ power quality is crucial due to the huge potential investment in preserving and developing utility grids by replacing with RES. The grid-connected RESs can make problems related to power quality for the power grid. Since the nature of RES, producing this type of energy is uncontrollable and accompanied by many fluctuations.

Increasing the penetration of RESs during times leads to serious concerns about power quality. On the other hand, disturbances of the grid like voltage sag generated by either short circuit faults or variations in frequency due to changes in amounts of production and consumption may make the operation analysis of grid connected RESs more complicated.

Recently, DGs and microgrids have been the research subjects of many authors. The grid connected inverters employed in DGs and microgrids are an interface between RES, energy storage systems (ESS), and the power grid. In order to decrease the investment and maintenance costs and reduce the cost of inverters in DGs and microgrids, multi-functional grid-connected inverters (MFGCI) are utilized. The MFGCI is an advanced inverter that not only could be an interface between DGs and power grids but also could improve the point of common coupling (PCC) power quality. Besides, the structure of the grid-connected inverters is similar to power quality conditioners like static var compensators (SVCs), active power filters (APFs), and so on.

Generally, the capacity of the grid-connected inverters is more than the capacity of the installed photovoltaic (PV) inverters and individual wind turbines. Consequently, it is able to adapt itself to intermittent and random wind speed and irradiation features. In addition, the grid-connected inverters may not consistently function at their full rated capacity. So, their extra capacity is available in their many operation times and can be used for PCC power quality enhancement. Thus, no extra power quality conditioner is required for an inverter based microgrid. However, power quality improvement is only one of the MFGCI's duties. So, the MFGCI's capacity for improving the power quality may be restricted, with the majority of its capability directed towards energy transferring from DGs. Hence, the suitable organization and optimized usage of their extra and valuable capacity are challengeable.

For power quality issues in the presence of neutral current, low power factor, load imbalance, harmonic components, and reactive energy demand, conventional MFGTI can operate as an APF and conduct current compensation [[1], [2], [3], [4], [5], [6]]. Based on Ref. [3], the grid interface inverter is used to perform important tasks including 1) Transferring the active power received from RES (wind, solar, etc.), 2) Supporting the load's required reactive power, 3) Compensating the current harmonics in the PCC, and 4) Compensating the neutral current and unbalanced current in a three-phase, four-wire grid. Furthermore, with proper control of the grid interface inverter, all four mentioned targets can be performed separately or simultaneously. The power quality limitations at the PCC are well maintained within the range of their operating standards without additional hardware costs. A robust control scheme is proposed in Ref. [4] without line voltage measurement for DG inverters with LCL filter, which 1) provides robustness active damping control function and is simple under the variations of grid and filter parameters; 2) eliminates the induced distortion from the grid without needing basic information about the distortion and unbalancing of the grid; 3) digitally maximizes the converter dynamic efficiency by an effective control function; and 4) provides dynamic response capability for active damping controllers and current tracking.

The instantaneous p-q-r theory based method is presented in Ref. [5] to remove the neutral current of the source with a four-wire compensator, a type of multi-functional power quality compensator. Also, these MFGCIs can compensate the power quality problems related voltage to like voltage swell/sag [[7], [8], [9], [10], [11]], unbalanced voltage [[7], [8], [9], [10],^12^], harmonics [13], and flicker [14]. In Ref. [7], a comprehensive research studies the PV inverter's performance under the compensation of unbalanced voltage sags. In particular, a control scheme is adopted to continuously plan the PV inverters performance in voltage sag scenarios by adjusting two control parameters. Therefore, power quality indicators like harmonic distortion and power ripple are regulated to be more precise than previous scalar approaches, where only discrete values are considered for these two parameters. On the other hand, three control algorithms are also developed to continuously calculate control parameters based on power ripple and harmonic distortion measures.

Since voltage sags may differ in terms of value and shape, different approaches are adopted to deal with these challenges. For instance, the method used in Ref. [8] is a flexible control design utilized in three-phase grid-connected inverters. In this method, when voltage sag is three phases and balanced, reactive power is fed by inverters so that the magnitude of voltage is boosted in individual phases. However, when single-phase or phase-to-phase faults occur, the inverter should provide a uniform voltage by decreasing the symmetric negative sequence component and removing abrupt voltage rises. So, over voltages and under voltages are prohibited, and the controller of [8] avoids interruptions in the system by supporting the desired voltage. Considering the limitations of the system, balancing system limitations, balancing these two issues is a mandatory policy.

In [9], a new current control design is presented to enhance the voltage source converter (VSC)-based STATCOM performance in the case of adverse unbalanced situations and faults. In this method, the major enhancement is to reduce the fault current negative sequences. If the current negative sequence is reduced, the apparent nominal power decreases; as a result, the cost of power equipment, including converters and power switches, reduces. In the control structure proposed in Ref. [9], the common synchronous frame based current controller is assumed for controlling the positive sequence voltage of the VSC output. This work is carried out by calculating the modulation index (MI) and the suitable output voltage angle (α) using dq components of the reference output voltage in the synchronous framework. An additional element is incorporated into the common vector current controller within this framework that regulates the negative sequence voltage of the VSC output. The manageable negative sequence voltage addresses the negative sequence line voltage's impact caused by imbalanced scenarios, consequently reducing the negative sequence current observed from the STATCOM side. The added component output results in an oscillation angle with a desired magnitude and phase. The output negative sequence voltage is then added to angle α [9].

Ref. [10] offers a novel approach to deal with the harmonics and reactive power. The strategy relies on a solar array side's grid-connected inverter and involves an optimization model to improve power quality. In this work, an improved current detection method called Conservative Power Theory is utilized to derive components of harmonics and reactive current from the load current. Additionally, the paper employs a hierarchical optimization model for power quality at the optimized PCC.

Ref. [11] introduces a control design for solar arrays to enhance the PCC's power quality while considering the inverter's size. Also, an adaptive neuro-fuzzy inference system (ANFIS) based MPPT control scheme is implemented in a two-phase interleaved boost converter, addressing the oscillation of dc-link voltage in the solar system. This controller considers the nominal size of the inverter.

A method is discussed in Ref. [12] for both harmonic filter current compensation and injected power by PV systems. The scheme observes the allowable output of the PV inverter and peak current. As the output PV power should be limited to a specific value, the paper also adopts a control design based on fuzzy logic.

Ref. [13] presents a multi-purpose hybrid strategy for a power quality converter that connects the main grid to the microgrids and integrates DG units with storage devices.

Ref. [14] provides a multi-functional voltage source inverter for properly injecting the PV active power output in the grid, supplying the demand, compensating reactive power requirements, dealing with harmonic components, and reducing the harmonic distortion level.

Multi-purpose grid-connected inverters play several interesting roles, including injecting energy output from renewable sources into the main grid, compensating reactive power and harmonics, and regulating voltage. To perform the former, i.e. injection of energy, reference current signals need to be produced. Ref. [15] designs several controllers to limit the magnitudes of currents while taking into account the power quality performance indicators at the PCC.

The authors in Ref. [16] analyze the performance of multi-purpose inverters in terms of reactive power injection. This work is used in PV systems and adopts a thermal model to present temperature predictions of power equipment.

The limited capacity issues of common MFGTIs are reviewed in Refs. [17,18]. An MFGTI can be considered as the interface of the RES in the power grid, and simultaneously deals with harmonics and reactive current in the microgrid as an auxiliary service. Therefore, the rated MFGTI size is limited to compensate the power quality.

The authors of [18] adopt the conceptual power quality evaluation algorithm by the analytical hierarchical theory to present two MFGCIs for optimizing the control goals. One objective function helps achieve the desired power quality by utilizing the minimum rated capacity of MFGTI. The other objective function concerns improving power quality and also examining the feasibility of certain conditions to achieve the available rated capacity.

In [17], the existing marginal capacity of MFGTI is taken into account, and a targeted compensation approach is suggested. Therefore, the corresponding power quality problem is restricted to issues of harmonic and reactive current. A controller is introduced [18] for effective use of MFGTI capacity, even though the MFGTI functions are limited to enhancing the power quality based on current in a single-phase system.

In [19], a flexible control method is proposed for electronic power converters that can be used as either a local power supply interface or an active power filter.

Ref. [20] employs impedance modeling to analyze the small-signal stability of an MFGTI exposed to an unbalanced load. To mitigate the impedance caused by the load, a compensation algorithm is proposed to modify the power quality of MFGTI using the conservative power theory (CPT), which includes stability analysis.

Ref. [21] introduces a novel arrangement of MFGTIs to solve voltage and current-based power quality issues irrespective of MFGTI capacity. The calculation method of the reference voltage and current for MFGTI control is presented in Ref. [21] by only subtracting the measured current or voltage from the rated value. Therefore, a research gap still exists in control and compensation approaches.

This study proposes a new MFGTI structure so that it can compensate most disturbances with only one multifunctional inverter. The proposed MFGTI can compensate both grid's and load's harmonic distortion in shunt compensation mode as a SAPF. The proposed control strategy of SAPF employs a Second order sequence filter (SOSF) to derive the positive-sequence components of the load non-sinusoidal current. Besides, by using a new hierarchical control with three layers, the MFGTI is capable of compensating unbalanced PCC voltages by decoupled dual synchronous reference frame (DDSRF) method in the shunt compensation mode. The details of DDSRF are completely discussed in the next sections. In addition, droop control is used to compensate the interruptions. This MFGTI is connected in series with the grid via a three phase injection transformer as SeAPF. In this series mode, the MFGTI can mitigate voltage sag/swell through the appropriate series compensation voltages. The proposed SeAPF strategy is based on damping PI controllers with a feedforward loop. Finally, an algorithm is proposed for a smooth transition between modes by considering the type of power quality problem that needs to be compensated. The main contribution can be summarized as follows.

• Using an SOSF based method to extract the harmonic current active components for individual three-phase systems through a straightforward sampling function;
• Proposing a damping PI controllers with feedforward loops for series compensation;
• Presenting a three levels hierarchical controller to compensate the imbalanced voltage and interruption;
• Suggesting a DDSRF based negative sequence extraction strategy for balancing the PCC voltage that can be located in third level of the hierarchical control;
• Providing an algorithm for a smooth transition between modes according to the type of power quality problem.

According to above discussions and the mentioned contributions, research gaps are as:

• Compensating the harmonic components, unbalanced voltage swell/sag, and interruption is rarely performed by only one inverter;
• A scheme is not yet offered for compensating the harmonics caused by nonlinear loads or grid distortions.

A preprint of this paper has previously been published [22]. The subsequent sections of the paper are structured in the following manner. The proposed multi-functional inverter model and control strategies for MFGTI are investigated in section 2. In section 3, state transition algorithm and its flowchart are presented. Section 4 presents simulation results for different cases of compensating by MFGTI. Eventually, section 5 concludes this paper.

## 2 Modeling of the proposed multi-functional inverter

The MFGTI structure is presented to improve power quality based on voltage, current and harmonics. The proposed MFGTI can be connected in series or shunt with the system via bidirectional switches. Power quality improvement by this structure can be achieved by using only one inverter without additional capacity design. Different active power injection compensation strategies are provided and different control designs for various working modes are presented. The shunt converter controller is used to compensate harmonics by SOSF based method. The series converter controller uses the damping PI controllers and feedforward loops to inject the desire reference grid voltage for balanced and unbalanced voltage sag/swell. And, a three level hierarchical droop control ((p/f) and (Q/v)) is incorporated to obtain suitable voltage and current signals to overcome the unbalanced voltage and interruption.

### 2.1 Proposed multi-functional series-shunt converter

**Fig. 1:**
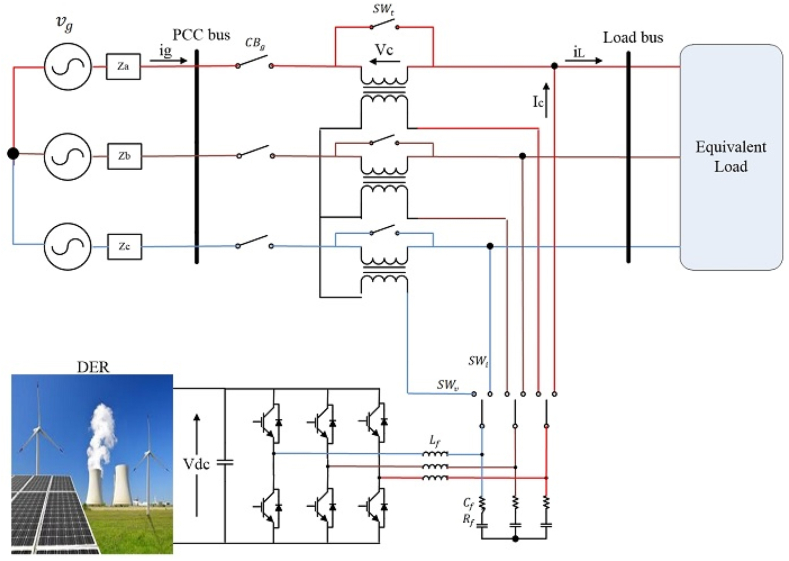
Proposed MFGTI configuration.

The proposed new MFGTI structure is shown in Fig. 1. The main difference between the proposed MFGTI in this paper compared to MFGTI presented in Ref. [21] is that series or shunt connection of the converter can be considered here to compensate the various grid power quality issues, including harmonics, while harmonic compensation is not considered in Ref. [21]. Bidirectional switches, (${SW}_{v}$) and (${SW}_{i}$), allows this converter to operate in various working modes based on switching functions and interruptions.

In Fig. 1, $v_{g}$ and $i_{g}$ represent the grid voltage and current, respectively. $Z_{a},Z_{b}\;and\;Z_{c}$ represent the grid impedances for each phase. The grid is considered to be a strong grid, having low short circuit impedance. $v_{c}$ and $i_{c}$ are consecutively compensation voltage and current for voltage adjustment. $L_{f}$, $C_{f}$ and $R_{f}$ are filter's inductance, capacitance, and damping resistance, respectively.

Table 1 provides four working conditions of MFGTI. In mode A, the MFGTI acts as a typical grid connected inverter, which converts the DC power produced by DER into the grid AC power. Current-based or shallow voltage-based power quality concerns along with current harmonics are compensated in mode B, where switches, ${SW}_{i}$ and ${SW}_{t}$, are off and ${SW}_{v}$ is on. The MFGTI injects compensation currents into the grid in shunt-connected to the grid.

**Table 1:** MFGTI's working modes and functions.

Mode	Configuration	${\text{sw}}_{t}$	${\text{sw}}_{i}$	${\text{sw}}_{v}$	Functions
A	Shunt	on	on	off	Traditional power flow control
B	Shunt	on	on	off	Current harmonic compensation
C	Series	off	off	on	Compensation of deep voltage sag/swell
D	Shunt	off	on	off	Uninterruptible power supply

Mode C occurs when the MFGTI is in series with the grid. In this case, the MFGCI supplies the necessary voltage to compensate for voltage-based power quality issues. The switches, ${SW}_{i}$ and ${SW}_{t}$, are on, while ${SW}_{v}$ is off.

It should be noted that the proposed MFGTI can compensate the unbalanced and balanced voltage swell/sag created by the grid, as well as the current and voltage harmonics. In this way, it eliminates the current harmonics generated by the grid side's nonlinear load. Besides, it removes the harmonics across the linear load created on the grid side. Finally, in order to supply uninterrupted power for the load when the grid is disconnected, mode D occurs. In this condition, the switches, ${SW}_{t}$ and ${SW}_{v}$, are off, while ${SW}_{i}$ is on.

### 2.2 Compensation strategies of MFGTI

In this section, the compensation strategies and control methods of MFGTI are presented to regulate the PCC voltage and compensate the unbalanced and balanced voltage swell/sag along with the harmonics compensation. Compensation strategies in modes B and C, the case of the occurrence of voltage issues, rely on SOSF and DDSRF methods, respectively. Moreover, the MFGTI state variations algorithm is proposed to create a transient state from state B to C, considering the maximum power and permissible capacity of the converter.

#### 2.2.1 Voltage adjustment in shunt state (mode B)

Shunt active power filter (SAPF) is a harmonic reactive power source. A current equivalent to the harmonic current equal caused by the non-linear load is injected into the system by this SAPF. The MFGTI performs this in the PCC through an interface inductor ($L_{f}$). The form and amplitude of the compensation current are obtained once the load current ($i_{L}$) is measured. Then, a comparison with a sinusoidal reference current is performed.

The SAPF control strategies aim to produce a gate signal suitable for switching the VSC per the load's variations in voltage and current signals. The actual SAPF currents pursue instantly the reference currents, even if there is a random voltage flicker. By achieving a fixed voltage for the DC capacitor, there is no actual power exchange between the power system and the capacitor under any condition. So, the optimal performance of the filter is obtained, harmonics of the current and voltage signals at the PCC are mitigated and filtered out, and the THD is decreased at this bus up to an acceptable level.

**Fig. 2:**
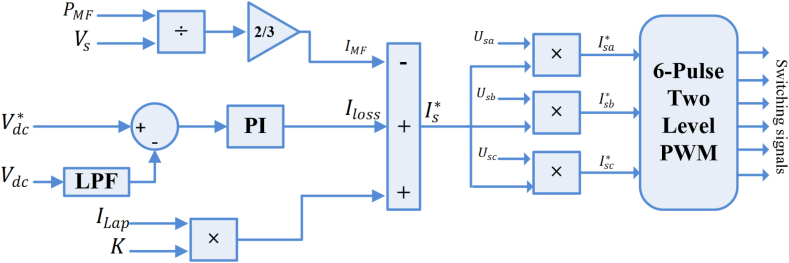
SAPF control block diagram.

**Fig. 3:**
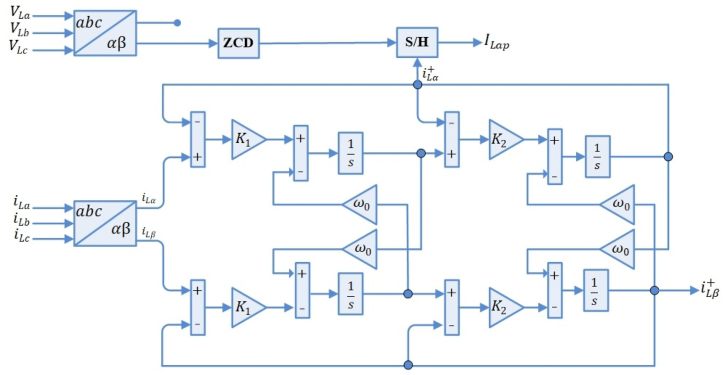
The second order sequence filter based extraction of load active components.

The control mechanism of a SAPF is presented in Fig. 2, wherein the positive-sequence components are extracted by a SOSF from the load's non-sinusoidal current. The configuration of the SOSF is illustrated in Fig. 3. These filters find predominant application for the extraction of positive-sequence components at the fundamental frequency in three-phase systems. The mathematical representation of the SOSF's open-loop transfer function is provided by (1).

(1) $$G_{sosf}\left(s\right)=\frac{K_{1}K_{2}}{\left(s-j\omega +K_{2}\right)\left(s-j\omega \right)}$$

Eq (1) can be characterized by its closed-loop transfer function as follows [23]:

(2) $${Gcl}_{sosf}\left(s\right)=\frac{K_{1}K_{2}}{\left(s-j\omega +K_{2}\right)\left(s-j\omega \right)+K_{1}K_{2}}$$

The structure entails two control coefficients of $K_{1},K_{2}$. A solution that balances the precision of FFPSC extraction with dynamic response can be offered by $K_{1},K_{2}$. The calculation process of this filter's coefficients is described in Ref. [24]. Assuming the utilization of SOSF helps in the load current FFPSC estimation. Consequently, through the current sampling at the load voltage's zero-crossing β-component, the magnitude of the load current's active component is determined. The load current sampled FPSC depends on the load current's active component amplitude since Clark transformation employs a fixed magnitude transformation in load signals. The appropriate current of the grid side would be the sought-after SAPF reference signal. This implies that balanced reference signals with a unity power factor should be obtained. The three main active components of the grid-side current magnitude are: grid-side current generated by the equivalent fundamental active power component of load at a nominal voltage of the PCC ($I_{Lp}$), the MFGTI ($I_{MF}$) associated grid-side current in SAPF mode, the semiconductors switching based losses, filters' power losses and so on, that are expressed as [23]:

(3) $$I_{s}^{*}=I_{Lp}-I_{MF}+I_{loss}$$

The load active power associated equivalent grid-side current is written as [23]:

(4) $$I_{Lp}=K\times I_{Lap}$$

Where

(5) $$K=\frac{V_{L}^{*}}{V_{s}}$$

$V_{L}^{*}$ represents the amplitude of load's reference voltage and $V_{s}$ shows the amplitude of the PCC voltage.

The MFGTI system power based current at grid-side is then expressed as [23]:

(6) $$I_{MF}=\frac{2}{3}\frac{P_{MF}}{V_{s}}$$

where $P_{MF}$ represents the system power of the MFGTI.

For regulating and maintaining of the MFGTI system's DC-link voltage in the SAPF mode, a PI controller is considered which leads to the filter's and converter's losses. The performance of controller at the DC-link voltage can be expressed as [23]:

(7) $$i_{loss}\left(n\right)=I_{loss}\left(n-1\right)+K_{p}\Delta e_{vdc}\left(n\right)+K_{i}e_{vdc}\left(n\right)$$

where $I_{loss}$ represents the losses in the MFGTI-SAPF system and output of the PI controller. $K_{p}$ and $K_{i}$ are the PI controller gains and $\Delta e_{vdc}$ shows the generated error in the DC-link voltage due to the difference between the current and previous sample times. $e_{vdc}$ is the voltage in the DC-link error. Next, the assessment of the amplitude of PCC voltage ($V_{s}$) and its in-phase templates is conducted. Subsequently, to provide instantaneous reference signals of SAPF ($i_{sa}^{*},i_{sb}^{*},i_{sc}^{*}$), it is multiplied by its reference amplitude. To obtain the pulses for SAPF switching, these references are sent to a two-level sine-pulse width modulation block (2L-SPWM).

#### 2.2.2 Voltage regulation in the series state (mode C)

**Fig. 4:**
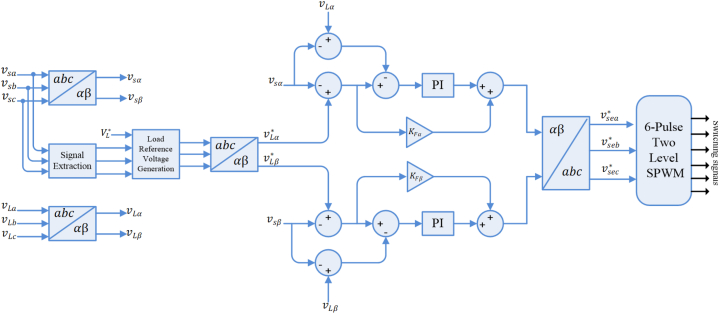
SeAPF control configuration.

Series active power filter (SeAPF) helps in compensation of voltage oscillations, including voltage sag/swell. In this filter, the voltage error that exists in the grid is calculated. Fig. 4 demonstrates the control scheme of SeAPF in α-β domain. The aim of SeAPF controller is making in-phase voltages at PCC and load. Equations (8), (9), (10) express the instantaneous reference voltages of load [23].

(8) $$v_{La}^{*}=V_{L}^{*}\times u_{sa}$$

(9) $$v_{Lb}^{*}=V_{L}^{*}\times u_{sb}$$

(10) $$v_{Lc}^{*}=V_{L}^{*}\times u_{sc}$$

Then, α-β domain is considered to convert the reference voltages of load. An integrated feed-forward and proportional damping controllers is employed for the α-β components to control the SeAPF. The α component of SeAPF reference voltage ($v_{se\alpha }^{*}$) is obtained as follows [23]:

(11) $$v_{se\alpha }^{*}=v_{L\alpha }^{*}-v_{s\alpha }$$

The SeAPF voltage ($v_{se\alpha }$) is obtained as (12) [23].

(12) $$v_{se\alpha }=v_{L\alpha }-v_{s\alpha }$$

A typical damping PI controller is considered for the error between $v_{se\alpha }^{*}$ and $v_{se\alpha }$ and its output is augmented to the output of the feed-forward path. The control signal's main component is also obtained by this path, while the drop in the filter circuit's voltage is compensated by the PI controller; this drop is not taken into the reference calculation accounts. Similar action would be employed for controlling in β domain. To use the α-β control signals in the stationary frame, another transformation is utilized. To produce SeAPF switching pulses, the obtained signals will be then sent to the two-level SPWM modulator.

#### 2.2.3 Hierarchical control design (mode A and D)

In this section, common P/f and Q/V controllers are hierarchically designed and implemented for A (load reference power supply and unbalanced voltage compensator) and D (interruption) modes. In addition, a negative sequence voltage compensator is proposed in control layer 3 to balance the load voltage. This model is designed in a rotating reference frame by controllers in three levels. Various parts of the hierarchical controller subsystems include first level of voltage and current control, second level of the droop control, and third level of unbalanced voltage compensation control.

A. Voltage and current controllers (level 1)

The voltage and current controller loops in level 1 are designed in the stationary reference frame. The proportional resonance (PR) controllers are implemented in the following transfer functions [25].

(13) $$G_{v}\left(s\right)=k_{pv}+\sum_{h=1,5,7}\frac{2k_{ih}\omega_{c}s}{s^{2}+2\omega_{c}s+{\left(2\pi hf\right)}^{2}}$$

(14) $$G_{i}\left(s\right)=k_{pi}+\sum_{h=1,5,7}\frac{2k_{iIh}\omega_{c}s}{s^{2}+2\omega_{c}s+{\left(2\pi hf\right)}^{2}}$$

where $k_{PV}$ and $k_{pi}$ denote the proportional coefficients; $\omega_{c}$ is the cutoff frequency to control the resonance bandwidth; $k_{ih}$ and $k_{iIh}$ are consecutively resonance controller gains of the $h^{th}$ order harmonic component of voltage and current (the main component harmonic is also included). The aforementioned level 1 controller is illustrated in Fig. 9.

B. Droop controller (level 2)

**Fig. 5:**
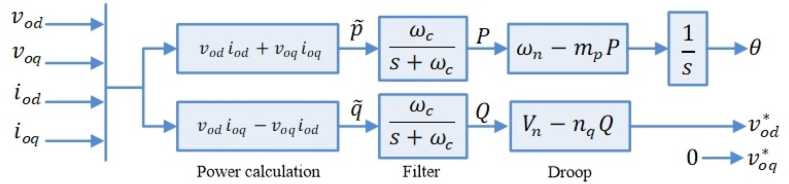
Conventional droop control.

The droop control aims to emulate the synchronous generator's governor. In a standard grid, synchronous generators handle load changes by changing the frequency based on the governor's droop characteristic. The same procedure can be implemented in inverters to increase/decrease frequency corresponding to the load variations. Similarly, reactive power is exchanged by creating a droop characteristic in the voltage magnitude. According to Fig. 5, the instantaneous components of active and reactive power ($\tilde{p}$ and $\tilde{q}$) are found through the output voltage and current as follows [25].

(15) $$\left\{ \begin{array}{c} \tilde{p}=v_{od}i_{od}+v_{oq}i_{oq}\\ \tilde{q}=v_{od}i_{oq}-v_{oq}i_{od} \end{array}\right.$$

The instantaneous power components are fed to a low-pass filter (LPF) to achieve active power P and reactive power Q related to the main component. The cutoff frequency of low pass filters ($\omega_{c}$) expresses the following relationship [25]:

(16) $$P=\frac{\omega_{c}}{s+\omega_{c}}\tilde{p},Q=\frac{\omega_{c}}{s+\omega_{c}}\tilde{q}$$

The active power control in the MFGTI is achieved by creating an artificial droop in the frequency of the inverter according to (17). The frequency ω is adjusted based on the droop gain ($m_{p}$) and the phase can be obtained by integrating the frequency. This behavior imitates the characteristics of the governor and the inertia of standard generators and generates some negative feedback. For example, provided that the generator's power output escalates, voltage phasors' recovery will slow down, and its angle will lag. In Eq. (17), $\omega_{n}$ is the rated reference frequency, while α is the inverter reference frame angle observed from a rotating reference frame at frequency $\omega_{n}$. Based on Eq. (17), the voltage angle of the inverter α varies corresponding to the real power flowing with a negative sign, and its gain is set as [25],

(17) $$\left\{ \begin{array}{c} \omega =\omega_{n}-m_{p}P\;\\ \dot{\theta }=\omega ,\theta =\omega_{n}-\int m_{p}Pdt\\ \alpha =-\int m_{p}Pdt,\dot{\alpha }=-m_{p}P \end{array}\right.$$

For controlling the inverter's reactive power, Eq. (18) adopts a droop in the voltage magnitude. Here, $V_{n}$ is considered as the d-axis reference nominal output voltage. The control design makes the d-axis of the inverter reference frame and the output voltage's reference magnitude in the same direction, and the q-axis reference is considered equal to zero [25].

(18) $$E=v_{od}^{*}=V_{n}-n_{q}Q,v_{oq}^{*}=0$$

Droop gains $m_{p}$ and $n_{q}$ are calculated by applying (19) for the desired frequency and voltage magnitude [25]:

(19) $$m_{p}=\frac{\omega_{\max}-\omega_{\min}}{P_{\max}},n_{q}=\frac{V_{od\_max}-V_{od\_min}}{Q_{\max}}$$

According to previous discussions, to form a comprehensive model in a standard reference frame, the reference frame of the MFGTI inverter is considered as the standard frame. To convert variables from the inverter reference frame to a standard frame, the angle δ for the MFGTI inverter is defined according to (20). It is worth noting that this angle δ expresses the angle between the reference frame of the MFGTI inverter and the standard reference frame [25].

(20) $$\delta =\int \left(\omega -\omega_{com}\right)$$

Now, to represent the system more simply, the voltage and current components of *dq* axes in (21) are combined to form vectors [25]:

(21) $$\left\{ \begin{array}{c} v_{odq}^{*}={\left[\begin{array}{cc} v_{od}^{*} & v_{oq}^{*} \end{array}\right]}^{T},i_{ldq}={\left[\begin{array}{cc} i_{ld} & i_{lq} \end{array}\right]}^{T}\\ v_{odq}={\left[\begin{array}{cc} v_{od} & v_{oq} \end{array}\right]}^{T},i_{odq}={\left[\begin{array}{cc} i_{od} & i_{oq} \end{array}\right]}^{T} \end{array}\right.$$

C. Unbalanced voltage compensation (level 3)

The suggested unbalanced voltage compensation is provided through the DDSRF for extracting the voltage and current negative sequence components of the MFGTI. Then, a two-loop control based on PI controllers is presented to produce the negative sequence reference voltage. The respective negative sequences in the dq synchronous frame are considered zero to balance the voltage of MFGTI. Finally, the negative sequence three-phase reference voltages in level 3 and the signal of voltage control in level 3 are added, then sent to the PWM block as shown in Fig. 9.

I. Dual synchronous reference frame

A general approach for regulating grid-connected and islanded converters involves the utilization of synchronous dq current controllers. The present methodology is founded upon controllers dependent on the rotation of reference frames and the introduced frequency component synchronization. One of the primary benefits of utilizing the synchronous reference frame (SRF) is the ability to convert the measured alternating currents and voltages in the correct sequence into a direct current amplitude within the dq frame via the park transformation method. It is suggested that conventional control techniques can be employed to attain optimal performance by designing a PI controller, provided that DC amplitudes are present [26].

(22) $$e^{-i\theta }=\left(\begin{array}{cc} \cos\left(\theta \right) & \sin\left(\theta \right)\\ -\sin\left(\theta \right) & \cos\left(\theta \right) \end{array}\right);J\left(\begin{array}{cc} 0 & -1\\ 1 & 0 \end{array}\right)$$

**Fig. 6:**
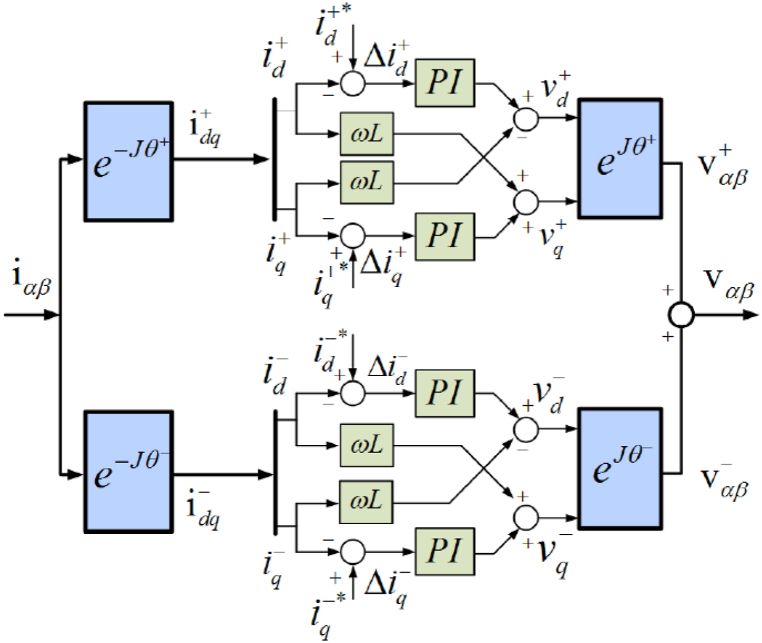
Double synchronous reference frame diagram.

Consequently, in imbalance voltage conditions in a three-phase power system, a negative sequence voltage component manifests, and regulating the negative sequence current may become imperative. In this scenario, it may be necessary to utilize a pair of synchronous dq controllers, with their respective angular positions corresponding to each sequence denoted by symbols sequence ($\theta^{+}$ and $\theta^{-}$). The aforementioned configuration is commonly referred to as DSRF, as illustrated in Fig. 6. The coefficient ωL in the given diagram represents the term decoupled in the dq domain, where L is the MFGTI system's output inductance. This analysis does not consider other harmonic components, as stated in Ref. [26]. The expression for the unbalanced voltage in an MFGTI is formulated as follows [26]:

(23) $$V_{g\alpha \beta }=v_{g\alpha \beta }^{+}+v_{g\alpha \beta }^{-}=V_{g}^{+}\left(\frac{\cos\left(\omega t+\varphi^{+}\right)}{\sin\left(\omega t+\varphi^{+}\right)}\right)+V_{g}^{-}\left(\frac{\cos\left(-\omega t+\varphi^{-}\right)}{\sin\left(-\omega t+\varphi^{-}\right)}\right)$$

where $\theta^{+}$ and $\theta^{-}$ are expressed as follows [26]:

(24) $$\begin{array}{l} \theta^{+}=\omega t+\varphi^{+}\\ \theta^{-}=-\omega t+\varphi^{-} \end{array}$$

Likewise, the vector of current is characterized as [26]:

(25) $$I_{\alpha \beta }=i_{\alpha \beta }^{+}+i_{\alpha \beta }^{-}=I^{+}\left(\frac{\cos\left(\omega t+\delta^{+}\right)}{\sin\left(\omega t+\delta^{+}\right)}\right)+I^{-}\left(\frac{\cos\left(-\omega t+\delta^{-}\right)}{\sin\left(-\omega t+\delta^{-}\right)}\right)$$

**Fig. 7:**
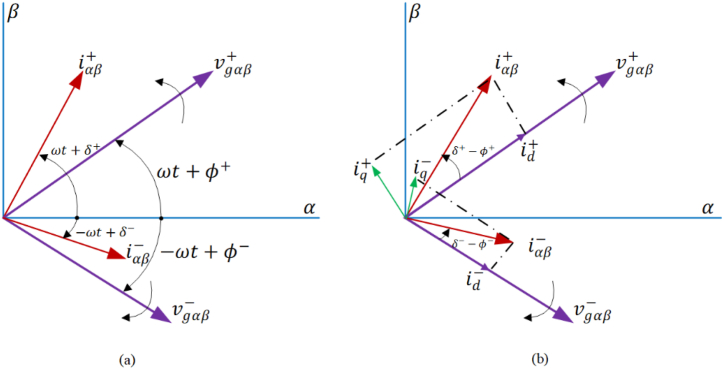
The MG output voltage and current respective positive and negative sequences. (a) MG positive and negative sequence voltage components and injected current, (b) the positive and negative sequence current dq components.

Fig. 7 illustrates the MFGTI's output voltage and current respective positive and negative sequences. Based on the associations above and Fig. 7a, it can be observed that the initial phase of the positive sequence voltage ($\varphi^{+}$) is not equivalent to that of the negative sequence voltage ($\varphi^{-}$). The dq current of positive sequence ($i_{dq}^{+}$) as presented in equation (20), is derived by implementing equation (26) and utilizing the angular position of positive sequence voltage ($\theta^{+}$) [26].

(26) $$\begin{array}{l} i_{dq}^{+}=e^{-i\theta^{+}}i_{\alpha \beta }=\left[\begin{array}{l} i_{d}^{+}\\ i_{q}^{+} \end{array}\right]+\left[\begin{array}{l} i_{d}^{-}\;\cos\left(\theta^{+}-\theta^{-}\right)+i_{q}^{-}\;\sin\left(\theta^{+}-\theta^{-}\right)\\ -i_{d}^{-}\;\sin\left(\theta^{+}-\theta^{-}\right)+i_{q}^{-}\;\cos\left(\theta^{+}-\theta^{-}\right) \end{array}\right]=\\ i_{dq}^{+}+e^{-j\left(\theta^{+}-\theta^{-}\right)}i_{dq}^{-} \end{array}$$

The dq current computation of the negative sequence ($i_{dq}^{-}$), as stated in equation (21), and shown in Fig. 7b, involves the utilization of equation (26) and the angular position of negative sequence voltage ($\theta^{-}$) [26].

(27) $$\begin{array}{l} i_{dq}^{-}=e^{-i\theta^{-}}i_{\alpha \beta }=\left[\begin{array}{l} i_{d}^{-}\\ i_{q}^{-} \end{array}\right]+\left[\begin{array}{l} i_{d}^{+}\;\cos\left(\theta^{-}-\theta^{+}\right)+i_{q}^{+}\;\sin\left(\theta^{-}-\theta^{+}\right)\\ -i_{d}^{+}\;\sin\left(\theta^{-}-\theta^{+}\right)+i_{q}^{+}\;\cos\left(\theta^{-}-\theta^{+}\right) \end{array}\right]=\\ i_{dq}^{-}+e^{-j\left(\theta^{-}-\theta^{+}\right)}i_{dq}^{+} \end{array}$$

Which we have in (26), (27) [26]:

(28) $$\begin{array}{l} i_{d}^{+}=I^{+}\;\cos\left(\delta^{+}-\varphi^{+}\right)\\ i_{q}^{+}=I^{+}\;\sin\left(\delta^{+}-\varphi^{+}\right)\\ i_{d}^{-}=I^{-}\;\cos\left(\delta^{-}-\varphi^{-}\right)\\ i_{q}^{-}=I^{+}\;\sin\left(\delta^{-}-\varphi^{-}\right) \end{array}$$

Cross-coupling in (26), (27) leads to producing an AC term with double frequency (2ω) in dq DC signals. The disparity between the angular positions of every synchronous frame governs the oscillation [26]:

(29) $$\begin{array}{l} \theta^{+}-\theta^{-}=\omega t+\varphi^{+}-\left(-\omega t+\varphi^{+}\right)=2\omega t+\Delta \varphi \\ \theta^{-}-\theta^{+}=-\omega t+\varphi^{-}+\left(\omega t+\varphi^{-}\right)=-2\omega t-\Delta \varphi \\ \Delta \varphi =\varphi^{+}-\varphi^{-} \end{array}$$

II. Decoupled DSRF

To attain the intended levels of injecting active and reactive power during grid disturbances, it is imperative to prevent oscillations in the measured dq currents, especially under imbalance scenarios and when employing the DSRF controller. The proposed DDSRF of [26] minimizes this adverse effect by estimating the 2ω oscillation amplitude and phase. Therefore, using a cross-decoupled network, such as (30), is feasible to generate the measured current through the independent positive and negative sequence reference frames. This strategy is similar to the method applied to PLLs in synchronization applications [26].

(30) $$\begin{array}{l} i_{dq}^{{+}^{\prime }}=i_{dq}^{+}+e^{-j\left(\theta^{+}-\theta^{-}\right)}i_{dq}^{-}-e^{-j\left(\partial \theta^{+}-\partial \theta^{-}\right)}i_{dq}^{-}\\ i_{dq}^{{-}^{\prime }}=i_{dq}^{-}+e^{-j\left(\theta^{-}-\theta^{+}\right)}i_{dq}^{+}-e^{-j\left(\partial \theta^{-}-\partial \theta^{+}\right)}i_{dq}^{+} \end{array}$$

**Fig. 8:**
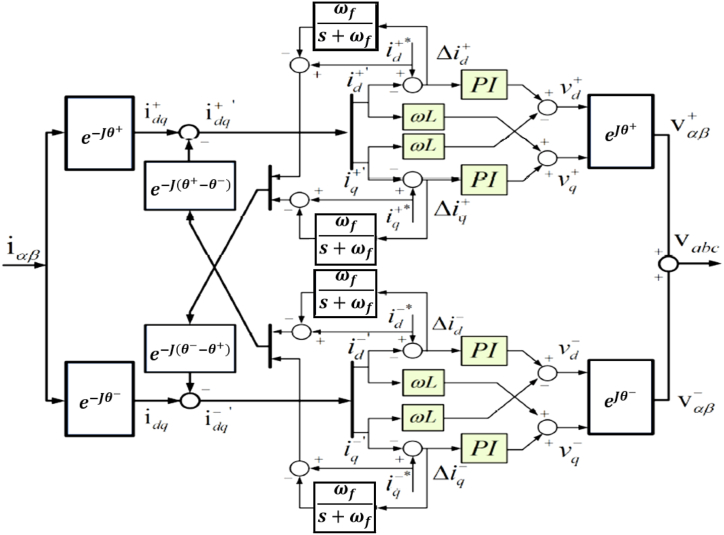
Decoupled dual synchronous reference frame diagram.

Fig. 8 illustrates the DDSRF controller application. Instead of filtering methods, the reference current provides the dq current mean value for the cross-decoupled term in (30). It is seen that the oscillation magnitude is equivalent to the opposite sequence dq current reference In steady-state, provided that the PI controller exhibits zero DC error. During the current errors, it is necessary to deduct the respective DC error from the reference current to achieve the current amplitude extraction value within the synchronous frame. Additionally, it is imperative to filter the aforementioned signal to prevent the occurrence of 2ω oscillations in the current error, as stated in reference. Upon amplitude estimation, the complete oscillation is determined by using park transformation to discern the angular position of both frames, which are obtained via a DSRF-PLL. The ultimate formulation for implementing DDSRF is presented below [26].

(31) $$\begin{array}{l} i_{dq}^{{+}^{\prime }}=i_{dq}^{+}+e^{-j\left(\theta^{+}-\theta^{-}\right)}i_{dq}^{-}-e^{-j\left(\partial \theta^{+}-\partial \theta^{-}\right)}\left(i_{dq}^{-*}\bar{.\Delta i_{dq}^{-}}\right)\\ i_{dq}^{{-}^{\prime }}=i_{dq}^{-}+e^{-j\left(\theta^{-}-\theta^{+}\right)}i_{dq}^{+}-e^{-j\left(\partial \theta^{-}-\partial \theta^{+}\right)}\left(i_{dq}^{+*}\bar{.\Delta i_{dq}^{+}}\right) \end{array}$$

**Fig. 9:**
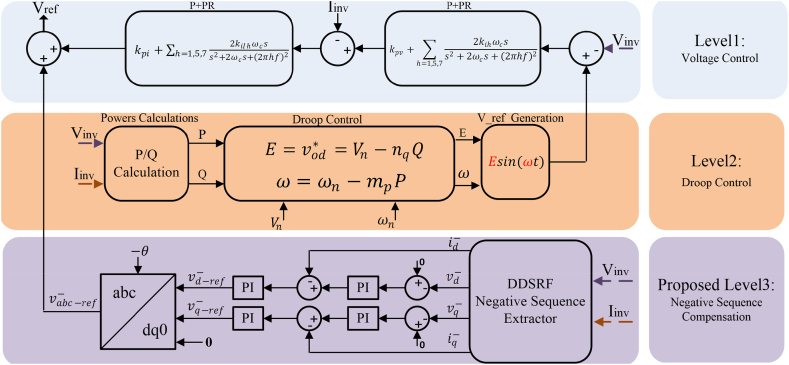
Droop controller integrated into negative sequence compensation controller.

A low pass filter (LPF) is unnecessary to achieve the current error mean value by high-value selection because there is no oscillation during steady-state conditions. This filter's cut-off frequency can be also set as follows [26]:

(32) $$\omega f=\omega /\sqrt{2}$$

III. Negative sequence reference voltage generation

In this paper, to produce the negative sequence reference voltage, a two-loop control at the third level is suggested, as depicted in Fig. 9. The negative sequences of reference voltage are augmented with the positive sequence reference voltage obtained by the voltage control signal in the third level of hierarchical control. The external voltage control loop of the negative sequence compensator includes the common PI controller, and the negative sequences of reference dq voltage are fixed at zero $V_{dq}^{-*}$. The output of the PI regulator is the same as the negative sequence reference current ($i_{dq}^{-*}$) for the current control loop. These reference values are compared with the currents extracted via DDSRF in the negative sequence, and the obtained signal of error passes from another PI controller. The respective output will be the same as the negative sequence reference voltage ($v_{dq-ref}^{-}$), added to the main reference voltage after transforming the inverse park with angle -θ. Fig. 9 shows the whole proposed control structure.

## 3 Switching scheme and transient states in MFGTI

**Fig. 10:**
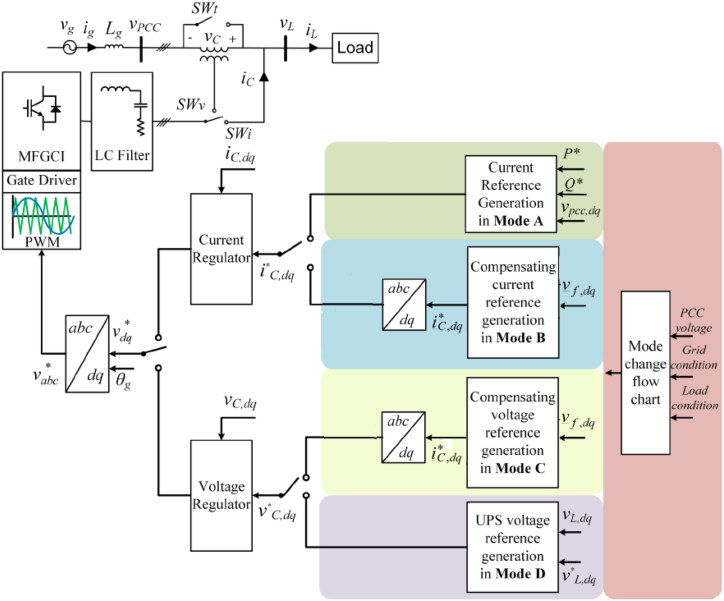
Overall control scheme for proposed MFGTI.

Fig. 10 illustrates the final control design of the MFGTI. This controller utilizes the measured values of MFGTI voltage, three-phase PCC voltage, and load's voltage and current. Afterward, these extracted currents and voltages are given to PI regulators. Then, by employing a second-order integrator with PLL, the measured values are transformed from the three-phase frame into the dq synchronous reference frame.

**Fig. 11:**
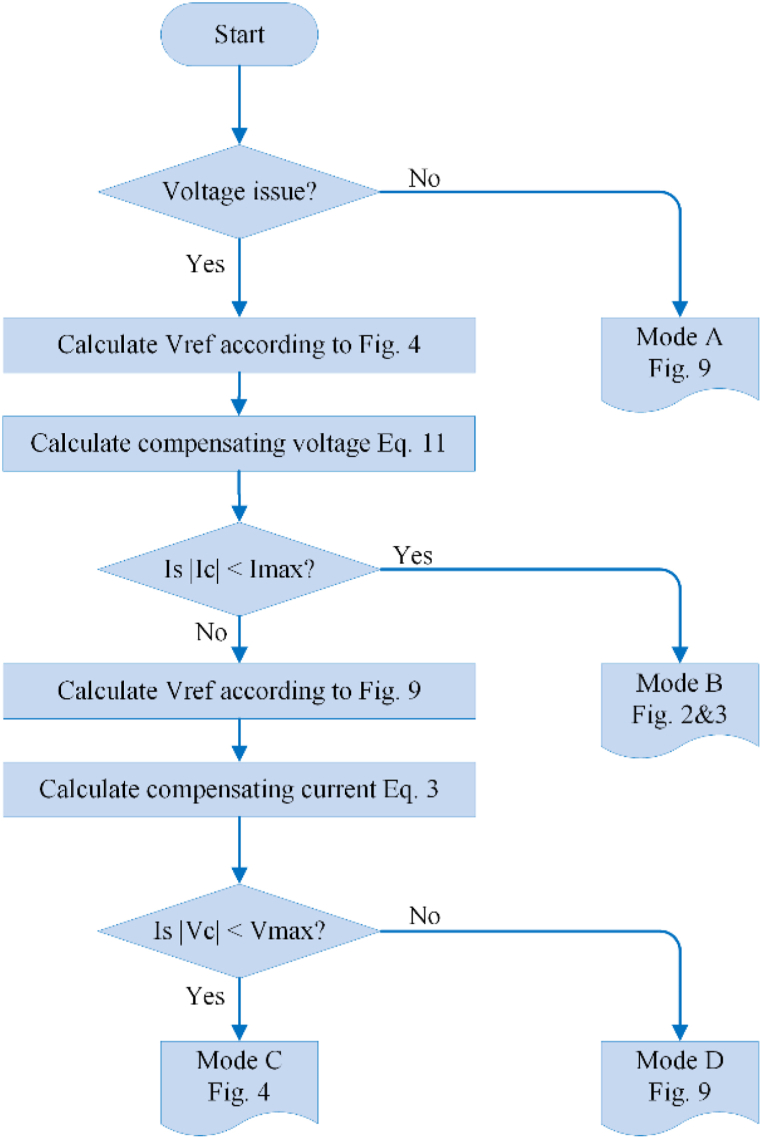
Flowchart of MFGTI mode switching for voltage regulation.

Determining the moment in which the MFGTI transfers from one state to another is significant. Fig. 11 shows the respective algorithm, assuming that the impedance of load and grid are previously determined.

The quality of PCC voltage can be identified by deriving the PCC voltage and current dq components. Voltage swell/sag issues are also detected based on variations in the RMS voltage amplitude. If there are no power quality problems associated with PCC voltage, the MFGTI operates in mode A as a conventional grid-tied inverter. When a fault occurs in the PCC, the algorithm checks the possibility of applying the voltage adjustment by the prescribed analysis according to the grid disturbances. When the compensation current is more than the maximum current of the MFGTI according to the inverter's available capacity, it switches from mode B to mode C for voltage adjustment. The reference load voltage corresponds to the grid voltage to transfer from the island mode (mode D) to the grid-connected mode (mode A).

## 4 The results and discussion

**Fig. 12:**
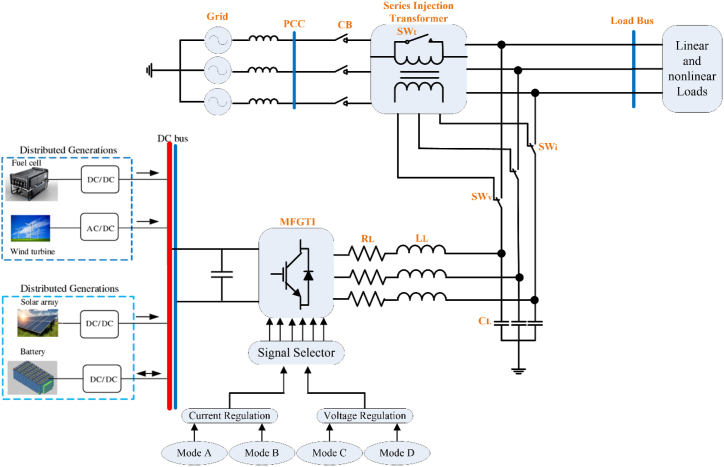
Overall scheme of proposed MFGTI for implementing.

In this section, the understudied grid is simulated in MATLAB/SIMULINK in the time domain to prove the robustness of the suggested MFGTI. By using the proposed method, most power quality problems, including unbalanced and balanced voltage swell/sag, harmonic components, and interruption are compensated. The overall design of the MFGTI for implementation is illustrated in Fig. 12. Two modes are considered for harmonic analysis and compensation, firstly, harmonic generating of non-linear load, and secondly, distorted and unbalanced source.

In the first case, a nonlinear load based on a three-phase diode bridge rectifier is used, which makes the grid current harmonic. In addition, the grid voltage is assumed with no harmonics and only suffers from balanced and unbalanced voltage sag/swell along with interruption. The proposed MFGTI strategy is able to firstly keep the load voltage constant at 1p.u by injecting the appropriate current and voltage and secondly, it compensates the current harmonics of the grid.

A three-phase linear load is used in the second case. But the source is distorted and unbalanced and causes harmonic pollution of load current and voltage, and also injects the negative sequence voltage. Even under this situation, the proposed method can compensate the harmonics at the load side, in addition to compensating unbalanced grid voltage sag/swell.

The series and shunt compensators used in this scheme operate under four conditions. In Mode 1 or Mode A, the compensator operates with the common droop controller (Q/v and P/f) corresponding to the hierarchical control discussed in Section 2, and it can deliver rated power to the load. In mode 2 or mode B, the compensator is used as a shunt active power filter that injects the load needed current and compensates the harmonic distortion by the SOSF method. In mode 3 or mode C, the compensator is connected in series with the active power filter where it can compensate the power quality problems associated with voltagelike swell/sag in voltage. Finally, in mode 4 or mode D, the control strategy of the MFGTI is the common droop control, which can supply the required voltage and power of the load under the condition of complete interruption and grid current disconnection. Fig. 11 illustrates the flowchart of changing the state between each mode. The required parameters related to the MFGTI are reported in Table 2.

**Table 2:** MFGTI parameters.

Parameters	Value
Grid voltage and frequency ($V_{g},f$)	400V,50Hz
Rated current and power of MFGTI ($S_{n},I_{n}$)	5kVA,10A
Grid Impedance ($R_{g},L_{g}$)	1Ω,1.5mH
Frequency droop coefficient ($m_{p}$)	$-2.612\times 10^{-6}$
Voltage droop coefficient ($n_{q}$)	0.005
SAPF coupling inductance ($L_{sh}$)	150mH
SeAPF RL filter ($L_{f\_se},C_{f\_se}$)	1.5mH,20μF
SeAPF PI coefficients	$k_{p}=0.7,k_{i}=3$
Voltage loop of DDSRF based negative sequence controller	$k_{p}=0.1,k_{i}=1$
Current loop of DDSRF based negative sequence controller	$k_{p}=5,k_{i}=15$
SeAPF Feed-Forward coefficient	$K_{FF}=1$
Second-order sequence filter coefficients	$K_{1}=100,K_{2}=250$

### 4.1 Grid harmonic current compensation caused by non-linear load

In this case, the switch related to the three-phase diode rectifier based nonlinear load is closed, and the switch related to the three-phase resistive load is opened, as presented in Fig. 12. Under the condition that the current of the grid is distorted, other phenomena like unbalanced and balanced voltage swell/sag along with load current disconnection occur according to the following conditions:

• At the t = 0–0.1s, mode A is activated, and the desired load is fed under the reference active and reactive powers at rated voltage and current.
• At the t = 0.1–0.2s, a voltage sag with a depth of 0.4p.u occurs in the source voltage. This will activate modes B and C at the same time.
• At the t = 0.2–0.3s, a voltage swell with a depth of 0.3p.u occurs in the source voltage. This will also activate modes B and C simultaneously.
• At the t = 0.3–0.4s, the voltage of the source is imbalanced so the voltage magnitudes of phases a, b, and c are equal to 1.3p.u, 0.7p.u, and 1p.u, respectively. Under these conditions, modes B and C are activated again to compensate the grid current's unbalance and harmonics by the DDSRF strategy.
• Finally, at the t = 0.4–0.5s, the grid current is completely disconnected, and the controller will go to the interruption compensation mode, i.e. mode D, to fully feed the load voltage and current by droop control.

**Fig. 13:**
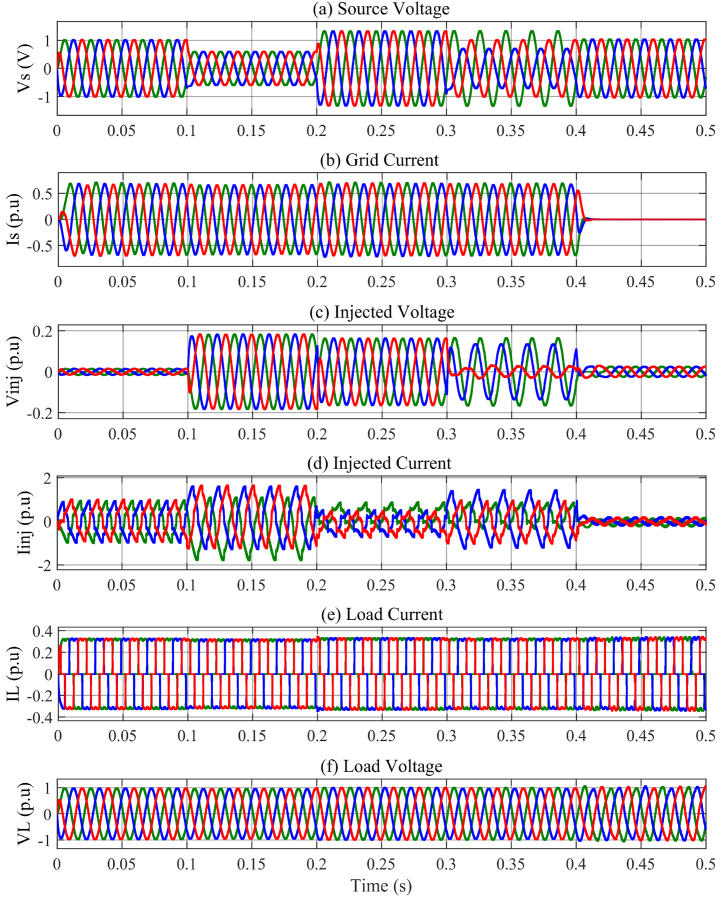
Results of grid voltage disturbances with harmonic load-first case. (a) Source voltage, (b) Source current, (c) Series injected voltage (mode B), (d) shunt injected current (mode C), (e) Nonlinear load current, (f) Load voltage.

**Fig. 14:**
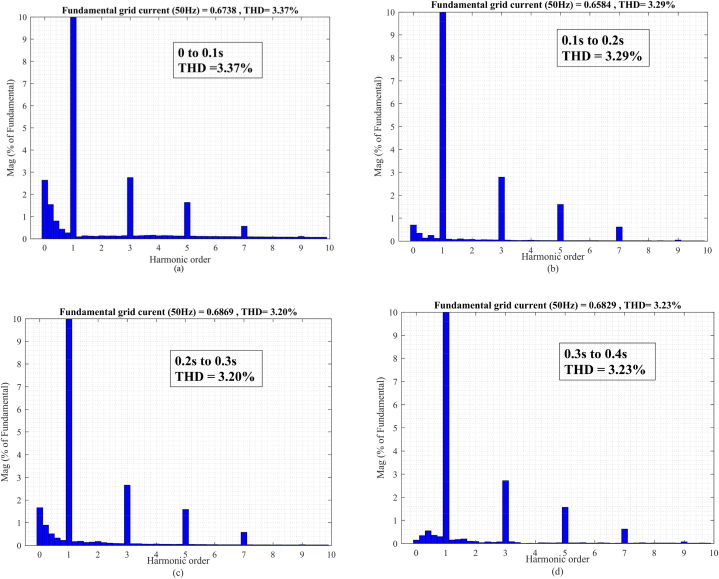
Harmonic distortion compensation of the grid current related to the first case. (a) 0–0.1s, (b) 0.1s–0.2s, (c) 0.2s–0.3s, (d) 0.3s–0.4s.

**Fig. .15:**
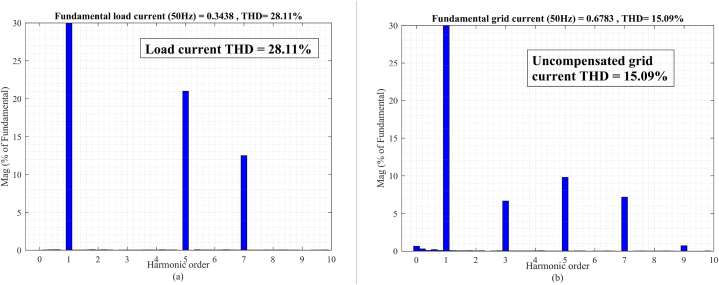
Harmonic spectrum of (a) nonlinear load and (b) uncompensated grid current in the first case.

In the $t_{0}=0s$ to $t_{1}=0.1s$, there is not any voltage problem as seen in Fig. 13a, therefore, the series injected voltage by the MFGTI approaches zero as shown in Fig. 13c. In this time interval, there is a harmonic issue due to the presence of nonlinear load. The distorted current of the nonlinear load is depicted in Fig. 13e. The THD of the nonlinear load is 28.11% as shown in Fig. 15a. So, the MFGTI injects a shunt compensating current of about 1p.u into the PCC. As a result, the THD of the grid current remarkably decreases based on Fig. 13b so the grid current THD is decreased to 3.73% from 15.09% as illustrated in Fig. 14, Fig. .15b.

During the $t_{1}=0.1s$ to $t_{2}=0.2s$, a voltage sag with a depth of 0.4p.u occurs in the source voltage in addition to the presence of the load harmonic components of the grid current. Simultaneously, to compensate the voltage sag, a three phase series voltage 0.2p.u is injected by a series transformer of the MFGTI in mode C. Also, a three phase shunt current 1.5 p.u is injected by MFGTI in the mode B to compensate the grid current harmonic as well as the portion of voltage sag.

Based on Fig. 13f, the load voltage remains constant at 1p.u during voltage sag. Furthermore, the grid current THD decreases from 15.09% to 3.29% as shown in Fig. 14b.

In the time interval of $t_{2}=0.2s$ to $t_{3}=0.3s$, a voltage swell with a depth of 0.3p.u happens in the source voltage. In order to maintain the load voltage at 1p.u, a series voltage opposite to the grid voltage with an amplitude of 0.18p.u is injected into the grid as illustrated in Fig. 13c. In addition, a shunt current 0.8 p.u is injected by MFGTI to compensate the grid current harmonics as shown in Fig. 13d. In this time interval, according to Fig. 14c, the grid current THD reaches 3.20% from 15.09%. As seen in Fig. 13f, the load voltage is fixed 1 p.u under voltage swell.

During $t_{3}=0.3s$ to $t_{4}=0.4s$, an unbalanced voltage sag occurs in the source voltages. In this state, the proposed DDSRF based negative sequence voltage compensation method is activated. Thus, according to Fig. 13c, injecting a three phase unbalanced series voltage by the series transformer of MFGTI leads to balance in the load voltages at the rated value (Fig. 13f). Besides, the shunt compensating currents is also unbalanced depending on unbalanced voltages of the grid (Fig. 13d). It should be pointed out that the Mode A, B, and C simultaneously operate under unbalanced voltage compensation. The grid current THD is decreased to 3.23% in this interval as depicted in Fig. 14d.

Finally, at $t_{4}=0.4s$ to $t_{5}=0.5s$, by disconnecting the grid current, the MFGTI controller goes to mode D to supply the load. In this mode, the proposed droop based hierarchical control is applied. During the interruption, the magnitudes of the load voltage and current remain unchanged at their nominal values.

According to Fig. 13, it can be seen that in all conditions, because of the presence of harmonic load, mode C, having the task of shunt compensation and injection of harmonic components opposite to the harmonics of the load, is constantly activated.

**Fig. 16:**
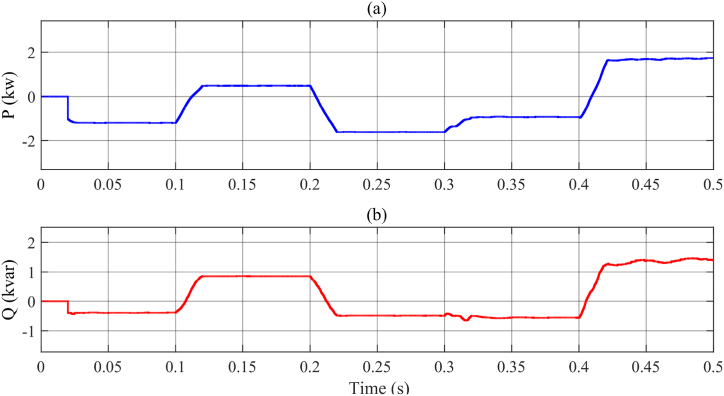
MFGCI injected powers with nonlinear load-first case. (a) Active power, (b) reactive power.

Fig. 16a and b give the active and reactive powers of the MFGTI exchanged with the system in the state, respectively, in while the nonlinear load is activated.

As illustrated in Fig. 16, in the interval of 0–0.1s, the extra active and reactive powers absorbed by the MFGTI are 1 kW and 500var, respectively. Then, in the 0.1s–0.2s, due to the voltage sag occurrence, the active and reactive powers fed to the grid are 500 W and 900VAR, respectively. Afterward, during 0.2s–0.3s, due to voltage swell, the active and reactive powers absorbed by the MFGTI are about 1.8 kW and 500VAR. Next, in the 0.3s–0.4s, since an unbalanced voltage sag occurs, generating the extra active and reactive powers increase up to 1 kW and 500Var which are absorbed by the MFGTI. Eventually, in the last interval, 0.4s–0.5s, due to an interruption in the grid currents, the MFGTI injects the active and reactive powers to the load by an amount of 1.8 kW and 1.5kVAR.

### 4.2 Harmonic compensation of load-side due to harmonic source

**Fig. 17:**
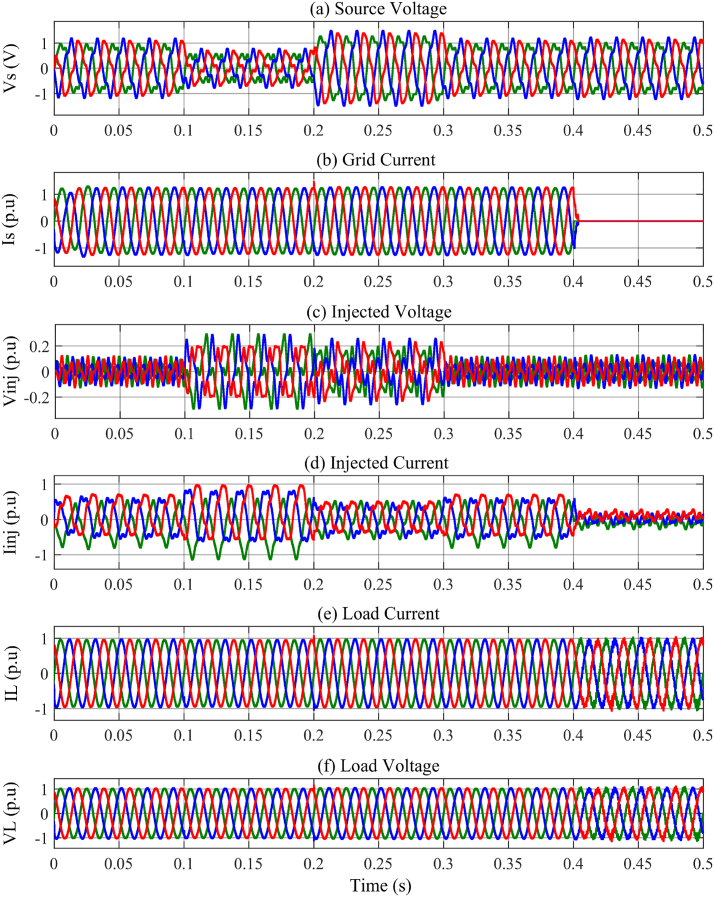
Results of grid voltage disturbances with resistive load and distorted grid (source) - second case (a) Source voltages, (b) Source currents, (c) Series injected voltages (mode B), (d) Shunt injected currents (mode C), (e) load currents, (f) Load voltages.

Under these conditions, a simple three-phase resistive load is used which is placed instead of the former nonlinear load. But the source is distorted in this case. In addition, voltage sag/swell and interruption also occur in the source and the grid similar to the previous case. In such situations, the proposed MFGTI strategy can keep the load voltage constant at 1p.u in all conditions caused by source disturbances in addition to the load current's harmonic compensation. Based on Fig. 17, the simulation results related to situations similar to subsection 4.1 are shown. The source voltage contains 3rd and 5th harmonics in all the mentioned conditions.

**Fig. 18:**
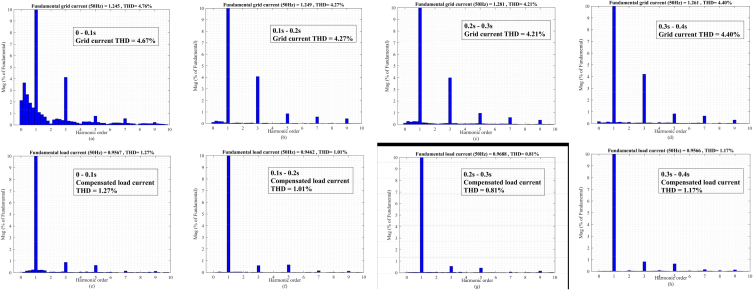
Harmonic spectrum of grid and load currents related to the second case under the various grid disturbances. (a)–(d) grid current THD, (e)–(h) compensated load current THD.

In the time interval of $t_{0}=0s$ to $t_{1}=0.1s$, there is a severe harmonic distortion in the grid voltages and those are relatively unbalanced based on Fig. 17a. According to Fig. 17b, prior to interrupt at t = 0.4s, the magnitude of source currents is balanced. Although voltages and currents of the grid are distorted, the THD of the load currents is only 1.27% by using the proposed MFGTI as shown Fig. 18e. Also, the grid current THD is 4.67% in this interval as illustrated in Fig. 18a. Due to load linearity, the load voltage THDs are the as same THDs of the load current.

The series injected voltage magnitude is 0.1p.u and it is distorted due to the grid voltage harmonics as Fig. 17c. The shunt injected current magnitude for compensating the load current harmonics is 0.5p.u as Fig. 17d.

In the $t_{1}=0.1s$ to $t_{2}=0.2s$, a voltage sag of 0.4p.u emerges in the source voltage in addition to a distortion. Since the grid voltage sag is unbalanced, the DDSRF method is enabled to balance the load voltage at the same time, as shown in Fig. 17c. The MFGTI injects an unbalanced series voltage so that the load voltages remain at 1 p.u. The THDs of the grid and load currents are 4.27% and 1.01%, as can be seen in Fig. 18b and f, respectively.

During $t_{2}=0.2s$ to $t_{3}=0.3s$, a voltage swell occurs in the source voltages, while they are distorted and unbalanced as shown in Fig. 17a. The currents of the grid are balanced and their THD is 4.21% as depicted in Fig. 18c. The series compensation currents are unbalanced and opposite to the grid voltages based on Fig. 17c. The shunt compensation current magnitude is 0.4 p.u to mitigate the load currents harmonics as shown in Fig. 17d. Furthermore, both THDs of load voltages and currents are 0.81% and the magnitude of the load voltages is balanced and equals to 1 p.u, as illustrated to Fig. 17, Fig. 18f, respectively.

The conditions for the $t_{3}=0.3s$ to $t_{4}=0.4s$ are similar to the first interval and it does not discussed again. The THD of the grid current is 4.40% as depicted in Fig. 18d. Also, in this interval, the THD of the compensated load current is 1.17% as shown in Fig. 18h.

In the $t_{4}=0.4s$ to $t_{5}=0.5s$, in spite of an interruption in the grid current, the linear load is supplied by the MFGTI under mode D so that both voltages and currents are kept at 1 p.u as shown in Fig. 17e and f. Although the load current THD is 4.5%, this value is within the IEEE allowable standard range.

All the above cases indicate that the suggested control scheme can effectively handle all voltage, current, and harmonic based power quality issues.

**Fig. 19:**
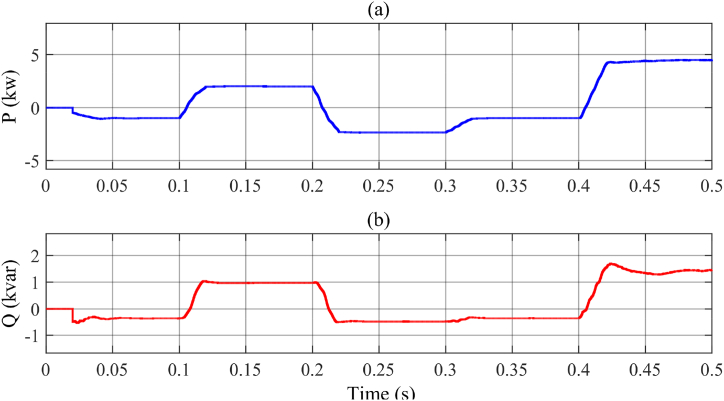
Injected powers by MFGTI with linear load and distorted grid. (a) Active power, (b) reactive power.

As depicted in Fig. 19a and b, in the time interval of 0–0.1s, the extra active and reactive powers absorbed by the MFGTI are 1 kW and 300var, respectively. Next, during the 0.1s–0.2s, according to the voltage sag occurrence, the active and reactive powers fed to the grid are 2.5 kW and 1kVAR, respectively. Afterward, during the 0.2s–0.3s, due to voltage swell, the active and reactive powers absorbed by the MFGTI are about 2.5 kW and 500VAR. Then, in the 0.3s–0.4s, since an unbalanced voltage sag occurs, generating the extra active and reactive powers increase up to1kW and 200Var which are absorbed by the MFGTI. Finally, in the last interval, 0.4s–0.5s, due to an interruption in the grid currents, the MFGTI injects the active and reactive powers to the load by an amount of 1.8 kW and 1.5kVAR.

## 5 Conclusion

An MFGTI was presented here for compensating the voltage, current, and harmonic power quality related problems. The MFGTI can compensate the mentioned power quality issues under four modes with a proper switching method between different modes. In modes A and D, reactive power-voltage (Q/V) and active power-frequency (P/f) droop characteristics based controller was used in a three layer hierarchical control to compensate the interruption and inject the load rated power. In mode B, the MFGTI acts as a shunt active power filter and a second order sequence filter based strategy was used for load and grid harmonic distortion compensation. In mode C, the proposed MFGTI acts as a series active power filter, and a method based on the damping PI controllers and the feedforward loops was used for compensation of sag/swell in the balanced grid voltage. For compensating the sag/swell in the unbalanced voltage, a strategy was presented according to the decoupled dual synchronous reference frame. In future work, we will explore the optimal capacity of MFGTI and will search for the voltage and frequency stability of a microgrid in the presence of MFGTI.

## Additional information

No additional information is available for this paper.

## CRediT authorship contribution statement

**Ehsan Akbari:** Conceptualization, Data curation, Formal analysis, Investigation, Methodology, Project administration, Resources, Software, Supervision, Validation, Visualization, Writing – original draft, Writing – review & editing. **Abbas Zare Ghaleh Seyyedi:** Conceptualization, Data curation, Formal analysis, Investigation, Methodology, Project administration, Resources, Software, Supervision, Validation, Visualization, Writing – original draft, Writing – review & editing.

## Declaration of competing interest

The authors declare that they have no known competing financial interests or personal relationships that could have appeared to influence the work reported in this paper.

## Nomenclature

$v_{g}:$: grid voltage
$i_{g},i_{L}:$: grid and load currents
$Z_{a},Z_{b},Z_{c}:$: grid phase impedance
$v_{c},i_{c}:$: compensation voltage and current
$R_{f}:$: filter damping resistance
$L_{f},C_{f}:$: filter inductance and capacitance
${SW}_{i}\text{:}$: switch of shunt APF
${SW}_{t}:$: switch of series transformer
${SW}_{v}:$: switch of series APF
$G_{SOSF}\left(s\right):$: open-loop transfer function of the SOSF
$G_{cl-SOSF}\left(s\right):$: closed-loop transfer function of the SOSF
$K_{1},K_{2}:$: control gains for SOSF method
s: laplace operator
$i_{LP}:$: grid-side current resulting from the load equivalent fundamental active power component
$i_{MF}:$: grid-side current of MFGTI
$i_{loss}:$: MFGTI's loss component
$P_{MF}:$: MFGTI's rated active power
$V_{s}:$: PCC voltage magnitude
$V_{dc}:$: DC-link voltage of MFGTI
$V_{L}^{*}:$: reference load voltage magnitude
$u_{sa},u_{sb},u_{sc}:$: PCC phase voltage
$i_{sa}^{*},i_{sb}^{*},i_{sc}^{*}:$: reference phase currents for shunt APF
$\Delta e_{vdc}:$: DC-link voltage error due to difference between the present and past sample times
$e_{vdc}:$: DC-link voltage error
$v_{se\alpha }^{*}:$: SeAPF reference voltage in α-axis
$v_{se\alpha }:$: SeAPF voltage α-axis
$K_{pv},K_{pi}:$: proportional gains of resonance controllers
$K_{ih},K_{iIh}:$: voltage and current gains of resonance controller
$h^{th}:$: harmonic order
$\omega_{c}:$: cut-off frequency of resonance controllers
$\tilde{p}:$: instantaneous active power of MFGTI
$\tilde{q}:$: instantaneous reactive power of MFGTI
$v_{od},v_{oq}:$: direct and quadrature compnents of the MFGTI's voltage
$i_{od},i_{oq}:$: direct and quadrature compnents of the MFGTI's current
$m_{p}:$: frequency droop gain
$n_{q}:$: voltage droop gain
$\omega_{n}:$: rated referece frequency
δ: angle between reference frame of the MFGTI and standard reference frame
$\theta^{+}:$: positive angular position
$\theta^{-}:$: negative angular position
$V_{g\alpha \beta }:$: unbalanced voltage of MFGTI in αβ-axis
$I_{\alpha \beta }:$: current vector of MFGTI in αβ-axis
$\varphi^{+}:$: positive sequence phase difference
$\varphi^{-}:$: negative sequence phase difference
$\omega_{f}:$: cut-off frequency of low pass filter
$v_{dq}^{-*}:$: reference negative sequence voltage in dq-axis
$i_{dq}^{-*}:$: reference negative sequence current in dq-axis

## References

[1] Liang Xiaodong (2017). Emerging power quality challenges due to integration of renewable energy sources. IEEE Trans. Ind. Appl..

[2] Zeng Zheng, Yang Huan, Zhao Rongxiang, Cheng Chong (2013). Topologies and control strategies of multi-functional grid-connected inverters for power quality enhancement: a comprehensive review. Renew. Sustain. Energy Rev..

[3] Singh Mukhtiar, Khadkikar Vinod, Chandra Ambrish, Rajiv K., Varma (2011). Grid interconnection of renewable energy sources at the distribution level with power-quality improvement features. IEEE Trans. Power Deliv..

[4] Mohamed, Yasser Abdel-Rady I., Seethapathy R. (2012). Robust line-voltage sensorless control and synchronization of LCL-filtered distributed generation inverters for high power quality grid connection. IEEE Trans. Power Electron..

[5] Sawant Rajendra R., Chandorkar Mukul C. (2009). A multi-functional four-leg grid-connected compensator. IEEE Trans. Ind. Appl..

[6] Wu Tsai-Fu, Shen Chih-Lung, Nein Hung-Shou, Li Guang-Feng (2005). A 1/spl phi/3W inverter with grid connection and active power filtering based on non-linear programming and fast-zero-phase detection algorithm. IEEE Trans. Power Electron..

[7] Castilla Miguel, Miret Jaume, Luis Sosa Jorge, Matas Jose, García de Vicuña Luis (2010). Grid-fault control scheme for three-phase photovoltaic inverters with adjustable power quality characteristics. IEEE Trans. Power Electron..

[8] Camacho Antonio, Castilla Miguel, Miret Jaume, Vasquez Juan C., Alarcon-Gallo Eduardo (2013). Flexible voltage support control for three-phase distributed generation inverters under grid fault. IEEE Trans. Ind. Electron..

[9] Babaei Saman, Bhattacharya Subhashish (2015). A control structure for PWM(2010)-controlled static synchronous compensators under unbalanced conditions and grid faults. Int. J. Electr. Power Energy Syst..

[10] Guo Wang, Xu Wu (2023). Research on optimization strategy of harmonic suppression and reactive power compensation of photovoltaic multi-functional grid connected inverter. Int. J. Electr. Power Energy Syst..

[11] Soumana Ricsa Alhassane, Juma Saulo Michael, Muriithi Christopher Maina (2022). New control strategy for multi-functional grid-connected photovoltaic systems. Results Eng..

[12] Soumana Ricsa Alhassane, Juma Saulo Michael, Muriithi Christopher Maina (2022). A new control scheme for limiting the compensation current and prioritizing power injection in multi-functional grid-connected photovoltaic systems. e-Prime-Adv. Elect. Eng. Elect. Energy.

[13] Bacon Vinícius Dário, Sérgio Augusto Oliveira da Silva, Josep M., Guerrero (2022). Multifunctional UPQC operating as an interface converter between hybrid AC-DC microgrids and utility grids. Int. J. Electr. Power Energy Syst..

[14] Karasala Chinna, Siva Kumar Ganjikunta (2021). An adaptive DC-link voltage control of a multifunctional SPV grid-connected VSI for switching loss reduction. IEEE Trans. Indus. Electr..

[15] Bonaldo Jakson Paulo, Tofoli Fernando Lessa, Arantes Monteiro Raul Vitor, Morales-Paredes Helmo Kelis (2021). Comparative analysis of techniques for the limitation of compensation currents in multi-functional grid-tied inverters. Int. J. Electr. Power Energy Syst..

[16] Callegari J.M.S., Silva M.P., De Barros R.C., Brito E.M.S., Cupertino A.F., Pereira H.A. (2019). Lifetime evaluation of three-phase multi-functional PV inverters with reactive power compensation. Elec. Power Syst. Res..

[17] Zeng Zheng, Yang Huan, Tang Shengqing, Zhao Rongxiang (2015). Objective-oriented power quality compensation of multi-functional grid-tied inverters and its application in microgrids. IEEE Trans. Power Electron..

[18] Bonaldo, Paulo Jakson, Helmo K., Paredes Morales, José Antenor Pomilio (2016). Control of single-phase power converters connected to low-voltage distorted power systems with variable compensation objectives. IEEE Trans. Power Electron..

[19] Jin Wei, Li Yongli, Sun Guangyu, Chen Xiaolong, Gao Yan (2018). Stability analysis method for three-phase multi-functional grid-connected inverters with unbalanced local loads considering the active imbalance compensation. IEEE Access.

[20] Zeng Zheng, Li Xiaoling, Shao Weihua (2018). Multi-functional grid-connected inverter: upgrading distributed generator with ancillary services. IET Renew. Power Gener..

[21] Choi W., Lee W., Han D., Sarlioglu B. (2018). New configuration of multi-functional grid-connected inverter to improve both current-based and voltage-based power quality. IEEE Trans. Ind. Appl..

[22] Akbari Ehsan, Abbas Zare Ghaleh Seyyedi (2023). Multifunctional power quality enhancement based on voltage and current in grid-tied microgrids with considering harmonics. Authorea.

[23] Devassy Sachin, Singh Bhim (2019). Implementation of solar photovoltaic system with universal active filtering capability. IEEE Trans. Ind. Appl..

[24] Xin Zhen, Zhao Rende, Mattavelli Paolo, Loh Poh Chiang, Blaabjerg Frede (2016). Re-investigation of generalized integrator based filters from a first-order-system perspective. IEEE Access.

[25] Pogaku N., Prodanovic M., Green T.C. (2007). Modeling, analysis and testing of autonomous operation of an inverter-based microgrid. IEEE Trans. Power Electron..

[26] Singh Bhim, Chandra Ambrish, Al-Haddad Kamal (2014).

